# Pre-transplant HbA1c and BMI are associated with long-term survival after lung transplantation

**DOI:** 10.3389/ti.2026.16741

**Published:** 2026-08-13

**Authors:** Zsofia Rosselli, Luca Saccarello, Andrea Macedo, Thomas Gaisl, Carolin Steinack, Fabrizio Pumo, Nail Atasayar, Georg Lang, Ömer Senbaklavaci, Isabelle Opitz, Irina Lea Dubach, Silvia Ulrich, Julian Müller, René Hage, Macé Matthew Schuurmans

**Affiliations:** 1 Division of Pulmonology, University Hospital Zurich, Zurich, Switzerland; 2 Faculty of Medicine, University of Zurich, Zurich, Switzerland; 3 Division of Thoracic Surgery, University Hospital Zurich, Zurich, Switzerland

**Keywords:** BMI changes, lung transplant, pre-transplant hba1c, risk assessment, survival benefit

Dear Editors,

Metabolic complications after lung transplantation are well recognized and are typically framed as downstream consequences of immunosuppressive therapy [1–3]. However, whether metabolic risk is already established before transplantation—and whether it is associated with post-transplant survival—remains insufficiently defined [3–6]. We therefore examined the association between pre-transplant metabolic parameters, specifically hemoglobin A1c (HbA1c) and body mass index (BMI), and long-term survival following lung transplantation, with a particular focus on the temporal stability of this association.

We conducted a retrospective cohort study including 257 consecutive adult lung transplant recipients transplanted at a single academic tertiary center between 2015 and 2024. The study was approved by the institutional review board (Req-2026-00435). Patients undergoing retransplantation were included at the time of their first transplantation and censored thereafter. Standard immunosuppressive therapy consisted of a calcineurin inhibitor, mycophenolate mofetil, and corticosteroids, with a center-wide transition from cyclosporine to tacrolimus during the study period [7]. Pre-transplant HbA1c and BMI were analyzed as continuous variables in relation to overall survival using Cox proportional hazards models adjusted for age, sex, transplant era, diabetes status and underlying lung disease. To explore temporal changes in predictive performance, time-dependent receiver operating characteristic analyses were performed at predefined intervals following transplantation.

During a follow-up period of up to 10 years, 81 deaths occurred, corresponding to a median survival of 8.5 years. Survival estimates were 89.7% at 1 year, 65.0% at 5 years, and 44.6% at 10 years. Among the 81 observed deaths, rejection-related causes were the most frequent (38/81, 46.9%), followed by infectious causes (23/81, 28.4%), cardiovascular (14/81, 17.3%), and malignancy-related deaths (5/81, 6.2%). Cause of death was missing in one patient. Mean HbA1c increased from 5.73% pre-transplant to 5.99% at 1 year, while BMI increased from 22.9 to 25.7 kg/m^2^, reflecting the expected post-transplant metabolic trajectory.

In multivariable Cox regression, pre-transplant HbA1c and BMI remained independently associated with mortality. Each 1% increase in HbA1c was associated with increased mortality risk (HR 1.41, 95% CI 1.08–1.80; p = 0.011), as was each 1 kg/m^2^ increase in BMI (HR 1.07, 95% CI 1.01–1.14; p = 0.033). Importantly, these associations persisted after adjustment for underlying lung disease, suggesting that differences between transplant indications alone are unlikely to fully explain the observed relationship.

The temporal behavior of this association provides additional insight. Time-dependent ROC analyses demonstrated modest discrimination of pre-transplant HbA1c for early post-transplant mortality, with an area under the curve (AUC) of 0.61 at 12 months after transplantation. However, predictive performance declined progressively over time, with AUC values of 0.53 at 36 months, 0.52 at 60 months, and 0.46 at 120 months, indicating limited long-term discriminatory ability. These findings suggest that pre-transplant glycemic status may provide prognostic information primarily during the early post-transplant period, while its predictive value diminishes during longer-term follow-up. Importantly, these findings should be interpreted as changes in predictive performance rather than evidence of true biological changes in metabolic risk over time, as formal longitudinal or time-varying effect modeling was not performed. Potential explanations, including cumulative immunosuppressive exposure, infection-related inflammation, rejection episodes, and progressive metabolic alterations such as weight gain and insulin resistance, remain hypotheses requiring further investigation. Accordingly, pre-transplant metabolic status may represent an early risk marker with diminishing discriminatory value over time rather than a fixed long-term predictor.

The observed divergence between metabolic burden and outcomes across immunosuppressive regimens provides an additional clinically relevant observation. Recipients receiving tacrolimus-based immunosuppression demonstrated higher observed survival compared with those receiving cyclosporine (HR 1.69, 95% CI 1.06–2.70, p = 0.029), despite less favorable metabolic profiles, including higher HbA1c levels (p = 0.0024) and greater weight gain (p = 0.0436). These findings illustrate the complex interplay between immunological efficacy and metabolic toxicity; however, they should be considered hypothesis-generating rather than evidence of a causal survival benefit. Residual confounding related to transplant era, evolving clinical practice, center experience, and factors influencing immunosuppressive selection cannot be excluded. [8]. Notably, HbA1c retained its prognostic significance after adjustment for diabetes status, suggesting that glycemic burden may provide additional risk information beyond a binary classification of diabetes. This observation is consistent with data from non-transplant populations demonstrating a continuous relationship between HbA1c and mortality, even below diagnostic thresholds for diabetes [9, 10]. Similarly, BMI contributed independently to risk, supporting the concept that metabolic risk is multifactorial and cannot be adequately captured by a single parameter.

From a clinical perspective, these findings support increased awareness of metabolic risk assessment before and after transplantation. Importantly, our study does not provide evidence regarding the effectiveness of specific interventions. Nevertheless, prospective studies evaluating optimization of glycemic control, nutritional assessment, physical rehabilitation, and weight management may help determine whether modification of metabolic risk can improve outcomes after transplantation. Future investigations should also evaluate emerging metabolic therapies, including incretin-based approaches, and structured lifestyle interventions in transplant populations before routine recommendations can be established.

Several limitations should be acknowledged. First, HbA1c and BMI represent only selected markers of metabolic status and do not capture the broader complexity of metabolic health, including lipid profile, insulin resistance, body composition, sarcopenia, nutritional status, and frailty. Therefore, the interpretation of metabolic risk in this study should remain restricted to the variables assessed. Second, the retrospective single-center design introduces the possibility of residual confounding. Although adjustment for clinically relevant covariates was performed, important factors influencing outcomes after lung transplantation, including perioperative severity, donor characteristics, primary graft dysfunction, rejection burden, infection burden, renal dysfunction, and functional status, were not fully captured.

Third, although post-transplant metabolic changes were described, formal longitudinal modeling was not performed, limiting causal inference regarding the interaction between baseline and evolving metabolic risk. Finally, the observational nature of this study prevents conclusions regarding whether targeted metabolic interventions can improve survival.

In conclusion, pre-transplant metabolic risk markers, specifically HbA1c and BMI, are associated with survival after lung transplantation. Their prognostic relevance appears strongest during the early post-transplant period. These findings should be interpreted as risk associations rather than evidence of causality, and further longitudinal studies incorporating comprehensive metabolic characterization are required to clarify mechanisms and determine whether targeted interventions can modify outcomes.

Sincerely.

## Data Availability

The original contributions presented in the study are included in the article/Supplementary Material, further inquiries can be directed to the corresponding author.
