# Supplementary material for: Pre-transplant HbA1c and BMI are associated with long-term survival after lung transplantation
Source: Transpl Int. 2026 Aug 13;39:16741. doi: 10.3389/ti.2026.16741 (PMC13518295; doi:10.3389/ti.2026.16741)
Supplement: Supplementary file 1 [file Table1.docx]

**Table Nr.1.** Demographic and Clinical Characteristics of Study Participants by Survival Status

|  | **alive (N=176)** | **dead (N=81)** | **ALL (N=257)** |
| --- | --- | --- | --- |
| **Age at TX** |  |  |  |
| Mean (SD) | 52.3 (13.3) | 51.0 (15.0) | 51.9 (13.8) |
| Median [Q1,Q3] | 56.1 [44.8,62.2] | 56.9 [43.1,62.4] | 56.7 [44.5,62.2] |
| **SEX** |  |  |  |
| Female | 81 (46.0%) | 29 (35.8%) | 110 (42.8%) |
| Male | 95 (54.0%) | 52 (64.2%) | 147 (57.2%) |
| **BMI** |  |  |  |
| Mean (SD) | 22.5 (4.17) | 23.6 (4.51) | 22.9 (4.30) |
| Median [Q1,Q3] | 22.2 [19.2,25.4] | 24.0 [20.2,27.4] | 22.6 [19.7,26.0] |
| **Diagnosis** |  |  |  |
| COPD | 88 (50.0%) | 34 (42.0%) | 122 (47.5%) |
| IPF | 43 (24.4%) | 26 (32.1%) | 69 (26.8%) |
| PAH | 5 (2.8%) | 4 (4.9%) | 9 (3.5%) |
| CF | 34 (19.3%) | 13 (16.0%) | 47 (18.3%) |
| Re-transplant | 6 (3.4%) | 4 (4.9%) | 10 (3.9%) |
| **TX_era** |  |  |  |
| 2015-2017 | 35 (19.9%) | 33 (40.7%) | 68 (26.5%) |
| 2018-2020 | 40 (22.7%) | 20 (24.7%) | 60 (23.3%) |
| 2021-2024 | 101 (57.4%) | 28 (34.6%) | 129 (50.2%) |
| **HbA1Cpre** |  |  |  |
| Mean (SD) | 5.66 (0.582) | 5.88 (0.935) | 5.73 (0.720) |
| Median [Q1,Q3] | 5.60 [5.30,5.90] | 5.70 [5.40,6.00] | 5.60 [5.30,5.90] |
| **HbA1cpost6mont** |  |  |  |
| Mean (SD) | 6.27 (1.44) | 5.76 (0.575) | 6.14 (1.29) |
| Median [Q1,Q3] | 5.90 [5.50,6.50] | 5.80 [5.40,6.10] | 5.80 [5.40,6.40] |
| **HbA1cpost1Y** |  |  |  |
| Mean (SD) | 5.99 (1.26) | 5.98 (0.939) | 5.99 (1.20) |
| Median [Q1,Q3] | 5.70 [5.40,6.20] | 5.80 [5.30,6.43] | 5.70 [5.40,6.20] |
